# Expressions of Individualization on the Internet and Social Media: Multigenerational Focus Group Study

**DOI:** 10.2196/20528

**Published:** 2020-11-04

**Authors:** Gwendolyn Mayer, Simone Alvarez, Nadine Gronewold, Jobst-Hendrik Schultz

**Affiliations:** 1 Department of General Internal Medicine and Psychosomatics Heidelberg University Hospital Heidelberg Germany; 2 Texas A&M University - Central Texas Killeen Texas, TX United States

**Keywords:** focus groups, discussion, qualitative research, generation, baby boomers, generation x, generation y, digital natives, identity, self, media use, internet research, social media

## Abstract

**Background:**

Growing individualization within the past decades has been described as a fundamental shift in society. Studies have reported how the digital age promotes new forms of individualism with self-tracking technologies and self-presentation in social networks. Potential harmful effects on the mental health of young adults have already been at the forefront of research. However, 2 questions that remain unanswered are how emotional experiences and expressions of self-relatedness differ among generations in their usage of the internet and social media, and if an increasing individualism can be observed by this.

**Objective:**

The aim of this study is to examine whether the use of the internet and social media has led people to be more concerned about themselves than former generations. The potential consequences of mental and emotional distress among different age groups are analyzed.

**Methods:**

A focus-group approach was chosen to study the following age groups: Baby Boomers (those born in 1950-1965), Generation X (those born in 1966-1980), and Digital Natives (those born in 1981-2000). We organized 6 focus groups with 36 participants who discussed their private usage of the internet and social media, different devices, platforms and functions, communication behavior, and self-tracking. We applied inductive category formation and followed the Standards for Reporting Qualitative Research (SRQR) checklist.

**Results:**

We found differences in the 3 studied generations regarding the reasons for their use of the internet and social media, the effects of this use, personal feelings and experiences, expressions of self-relatedness, and social relationships. The Baby Boomers voiced a wish to stay autonomous while being in contact with their personal network. Generation X included enthusiastic members who appreciate self-tracking for curiosity and fascination, as well as people who felt fears about data surveillance. The Digital Natives reported a wish to optimize their own body by self-tracking while being faced with norms and expectations that were communicated via the internet and social media.

**Conclusions:**

All generations expressed self-relatedness, yet by different means. The Baby Boomers expressed less individualism than Generation X and the Digital Natives, who felt the highest strain due to social comparisons. However, all generations reported specific, potentially problematic consequences for their mental health. Age-specific coping strategies are necessary to promote a mentally healthy way of using the internet and social media.

## Introduction

### Background

Individualization is considered to be a major transformation of postmodern society, and many efforts have been made to describe its reasons, preconditions, and consequences [1-3]. The beginning of its development started much earlier (likely with the courtly society of the Age of the Enlightenment [4]); however, most social science research focuses on the rise of individualization after World War II, characterized by an unprecedented number of choices opening up to individuals due to growing economic prosperity. Yet this freedom came along with uncertainty due to a loss of tradition, fragile norms, family structures, and gender roles [5]. The individual faced new challenges, such as having to plan one’s own personal life in the realms of education, marriage, or residence, which was formerly decided upon by one’s family or community [6]. This burden of choices has been linked to mental distress, with depression as its severest form [7].

Adolescents facing a modern paradigm of being as individualistic as possible have to cope with the increasing pressure of growing responsibilities. Being less tied to traditionally demarcated ways, they are now forced to make their own decisions at earlier ages than were former generations. Identity formation has become more than just a developmental step—it is a valuable resource needed for coping with the demands of individualization in a successful life [8,9].

Despite many theoretical approaches, a joint definition of the terms *individualization* and *identity,* as well as their interrelationships, is often missing. In this study, individualization is defined as an orientation of action that moves away from social or collective rules and toward person-based choices [10]. The precondition of this development is a stable and continuous feeling of being an individual, respectively, a *self* with an *identity* [11]. Role-theory describes the twofold nature of identity, with a social identity on the one hand, and a self-referential, personal identity on the other hand [12]. While personal identity results from one’s own experienced biography and gives one a feeling of uniqueness, social identity is built on affiliations and relationships with the social environment [13]. In turn, a constant sense of not fulfilling the expectations of others leads to an effort, which has been called *self-optimization* [14,15].

### Identity in the Digital Age

In the past 2 decades, mobile devices have allowed people to stay connected with others more than ever before. People are now interrelated in groups (eg, via apps such as WhatsApp) and permanently available at any place and time. This development impacts our identity in 2 ways: First, there is a technological acceleration of formerly long-lasting processes, whereby time pressure prevents us from keeping permanent, reliable relationships. As relationships are a constituting precondition of building up a stable, healthy identity, formerly lasting identities are transformed into “open, experimental, and fragmentary self-designs” [16]. Secondly, staying connected via electronic devices becomes part of our self-definition: “I share, therefore I am” [17] is the new credo, observed by clinical psychologist Sherry Turkle. However, in our real, nonvirtual relationships, valuable and real human interactions are lacking [18].

Microblogging services such as Twitter, Reddit, Tumblr, and audiovisual platforms like Instagram have opened up new perspectives on presenting oneself and being seen by the digital, generalized Other. A recent development of *storytelling* via Instagram and Snapchat, where the user presents a short collection of photos and videos in a narrative context by adding captions to the visual material [19], emphasizes this short-lived, quick, and anecdotal view on an otherwise small detail of a user’s life, as presented to one’s followers.

Research on these online services has focused primarily on mental health aspects, with some studies suggesting that social networking sites can be addictive [20]. Problematic internet use and addictive behavior have been related to loneliness [21,22], but there is a lack of differentiated data on individual social media services. A survey among German students also showed positive correlations with mental health variables, which were interpreted as a consequence of the positive effects of sharing photos with a community. In contrast, the “interaction on Twitter seems to be more impersonal and less likely to enhance a person’s social capital” [23] and thus correlates negatively with extraversion and self-esteem.

Despite a growing body of literature in this field, studies aimed explicitly at individualization are scarce and are mostly discussed within the context of narcissism [24], which is a psychopathological issue [25]. Many studies focus on the potentially harmful effects on young adults' mental health, and few include participants beyond the age of 30 years (such as in Chow and Wan’s study on Facebook depression [26]). Age-specific results are needed in order to give personalized recommendations for preventive measures regarding the mental health of a cross-linked, social-media–using population.

### The Quantified Self: Self-tracking and Self-optimization

Individualization has been reshaped recently due to a rising trend in the acquisition of personalized health information by wearable devices such as fitness trackers. This trend, which is often called *quantified-self movement* or *lifelogging*, is widespread, even beyond athletes. In 2016, the worldwide market reached up to 125 million devices, with estimates suggesting 237 million in 2020 [27]. Up to 69% of the US population regularly tracks at least 1 health indicator [28]. In Canada, a recent study found this number to be more than 66% [29]. The reasons for using self-tracking are, in most cases, increasing wellbeing or fitness, and sometimes, curiosity or the wish to question advice or a diagnosis delivered by a physician [30].

### Individualization and Internet Usage

Studies on the self and social identity run into the danger of remaining theoretical at the expense of personal experiences [31]. The concept of generational change is one possible way out of this situation, using the context of societal generations coined by sociologist Karl Mannheim in the 1920s [32]. This perspective encourages a focus on the shared cultural imprinting of a certain age group or cohort on the one hand and a differentiated look at single representatives of these groups at a psychological level on the other hand [33].

The Baby Boomer generation is characterized by “self-development, creativity, and pleasure of life” [34]. Regarding their usage of the internet at the beginning of social media development, Baby Boomers had the reputation of not understanding nonverbal cues in online communications such as emoticons [35]. However, Baby Boomers are now more familiar with social media and especially prefer forums and blogs for intellectual debates, entertainment, or sharing their own expertise [24].

The generation that followed the Baby Boomers, Generation X, is described as the “don't bother me“ generation, which has withdrawn into private life [34] after the political turbulence of the 1960s, which were run by the generation before. Research considered this generation to be the ”driving force behind“ new media developments, especially the internet [36]. Nevertheless, research focusing on the social media use of this generation remains scarce [37].

Following the ”X,“ the prominent Generation Y (also known as Millennials or Digital Natives, born between 1985 and 2000) are not committed to the values of former generations any longer, as they follow the mechanisms of ”egotactics“ [34]. This principle helps them ”make flexible decisions in everyday life at any time. They use a mix of self-reference and a sensitive, strictly opportunistic, tactile, and tactical behavior, exploring opportunities and developmental potential. Ideals, norms, and principles are of little help here“ [34]. This generation already grew up with the internet and does not remember a time before digitalization.

Many studies have described the changes caused by the internet and social media with respect to different age groups; however, how different generations experience the challenges and possibilities of the digital age, and whether there are any associations with different levels or degrees of individualization, has not yet been investigated.

### Research Questions

This study aims to determine whether or not the younger generation is more concerned about individualization, with self-optimization at its strongest form, and if the internet and social media have further promoted this development. A secondary objective is to detect potential consequences of mental and emotional distress among different age groups due to the permanent availability of information about others that allows for social comparisons. We look for generational differences in the usage of the internet and social media that go along with the emotional experiences and expressions of self-relatedness.

## Methods

### Study Design and Recruitment

This qualitative study employed focus group discussions for data collection. Focus groups were originally developed in media research to measure the influence of a certain stimulus, a ”focus,“ on an uninfluenced group [38]. This instrument has increased in popularity, especially in health research [39]. The reason lies in the ease of getting access to the perspectives of participants. Especially, ”not entirely encapsulated,“ nonreasonable responses can emerge in this unique form of communication [40].

We organized 6 focus groups, with at least 4 and a maximum of 8 participants from 3 consecutive generations of Baby Boomers, Generation X, and Digital Natives. We conducted 2 focus groups for each generation to collect adequate contributions for comparisons between the different age groups. We did not mix participants from different age groups since we wanted to facilitate a more relaxed and unbiased discussion flow [41]. All participants were of legal age.

Although the literature sets different limits for generations, we defined Baby Boomers as people born between 1950 and 1965, Generation X as those born between 1966 and 1980, and Digital Natives as those born between 1981 and 2000, following the definition of Palfrey and Gasser [42] in their famous publication, *Born Digital*.

The participants were recruited in Heidelberg University Hospital via public announcements, social networks (Facebook), and personal contact networks. They received a small monetary compensation for their participation.

Ethical approval for the study was granted by the Ethics Commission of the Medical Faculty of Heidelberg (S-039-2018) prior to recruitment. The Standards for Reporting Qualitative Research (SRQR) checklist was followed [43].

### Procedure of the Focus Group Discussions

All focus group discussions followed a standardized procedure. First, the moderator presented 5 pictures on a flipchart titled ”Me and My Devices.“ The pictures were of a smartphone, a tablet, a notebook, a desktop computer, and a smartwatch. The participants were asked to answer the question, ”Which of these devices do you use regularly in your private, everyday life?“ The first question had to be answered by every participant. The following questions could be discussed spontaneously in any order.

In the subsequent discussion, participants were initially asked about the importance they attached to the internet and mobile devices in their everyday life, how much time they spent with online media, and which platforms they usually visited. Further questions focused on communication, with whom and which groups it takes place, and if the usage of social media has changed communication and their respective social relationships in any way. The discussion flow was then directed to self-care and self-observation practices (ie, activities for self-tracking purposes, such as fitness apps, nutrition intake logs, or diaries with or without digital devices). The focus group guidelines are presented in Multimedia Appendix 1.

The first author (GM) moderated the focus groups, interfering only when the discussion lost its topic focus. The length of group discussions was between 76 and 82 minutes each.

### Data Analysis

All discussions were transcribed manually and analyzed by using MAXQDA (version 12; Verbi GmbH) [44]. Coding was carried out by the first author (GM), a psychologist with a background in medical sociology and experience in research on eMental health, and was supported by advice from a colleague with a background in psychosomatics.

The analysis followed the methodology of inductive category formation in order to identify patterns of experiences and interpretations [45,46], whereby the categories are not built on theoretical considerations but directly from the data itself, which allows for an unprejudiced assessment. The first step was to find a deductive criterion of selection to build categories and to define the abstraction level “in a manner that fits best to the research question” [46]. We chose a low abstraction level to get the best approximate of the feelings and personal observations of the participants. The deductive criterion of selection focused on personal experiences of the internet and social media and every expression of self-relatedness, which could provide information about individualization. For example, self-tracking was considered to be a subcategory of self-relatedness. Multiple codings were possible in order to respect the complexity of single expressions. The categories were built up step by step while working through the whole transcription. As a rule of thumb, 10-30 categories were recommended, and 10%-50% through the text, the category system was built, redundant categories were summarized, and the material was reviewed.

All main codes, their definitions, subcodes, and supporting quotes are listed in Multimedia Appendix 2. In the text, we have provided the frequencies of main codes and subcodes of all participants in brackets behind the respective category in order to illustrate the main differences regarding the relevance of the respective code in comparison to other codes. To maintain textual readability, we have not presented the differences between the generations in the text; however, they are displayed in Multimedia Appendix 2. Descriptive statistics were calculated for the data on participants' characteristics and their internet-capable devices. We report means and standard deviations regarding the age of the sample. In all other data types, we present frequencies and percentages.

### German Clinical Trials Register

This work is part of the study ”Between self-care and self-optimization: The impact of the internet and social media on the identity of mentally burdened and unburdened people in the different generations,“ which was registered by the DRKS with ID DRKS00014815.

## Results

### Participant Characteristics

The sample consisted of 36 participants born between 1955 and 1998: 11 Baby Boomers, 10 members of Generation X, and 15 Digital Natives. The mean age for the Baby Boomers was 60.55 (SD 2.66) years, 47.40 (SD 3.13) years for the members of Generation X, and 23.80 (SD 3.19) years for the Digital Natives (Table 1).

Nearly all participants owned a smartphone, except for 1 member of Generation X. A desktop computer was owned by the majority of Baby Boomers, while the other generations were more likely to own a notebook or a tablet. Of the 36 participants, only 4 had a wristband: 1 in the group of Baby Boomers and 3 members of Generation X (Table 2).

**Table 1:** Baseline characteristics of the participants (N=36).

Participant Characteristics			Values
**Age in years, mean (SD)**			40.46 (16.33)
	Digital Natives (born in 1988-1998)	23.80 (3.19)	
	Generation X (born in 1967-1976)	47.40 (3.13)	
	Baby Boomer (born in 1955-1962)	60.55 (2.66)	
**Gender, n (%)**			
	Male	17 (47.2)	
	Female	19 (52.8)	

**Table 2:** Participant ownership of internet-capable devices (N=36).

Device	Baby Boomers, n (%)	Generation X, n (%)	Digital Natives, n (%)	Total, n (%)
Desktop computer	7 (64)	3 (30)	3 (20)	13 (100)
Notebook	5 (45)	8 (80)	14 (93)	27 (100)
Tablet	5 (45)	7 (70)	6 (40)	18 (100)
Smartphone	11 (100)	9 (90)	15 (100)	35 (100)
Wristband	1 (9)	3 (30)	0 (0)	4 (100)
Total number of devices owned by each generational group	29 (2.64)	30 (3.00)	38 (2.53)	97 (2.69)

### Focus Group Discussions

By means of inductive category formation, 5 main themes of information were found: (1) reasons for the use of the internet and social media, (2) effects of the use of the internet and social media, (3) personal feelings and experiences, (4) self-relatedness, and (5) social relationships.

### Reasons for the Use of the Internet and Social Media

During the course of the discussions, all participant statements that provided insight into the causes and occasions of internet and social media use in daily life were coded with the main code, *Reasons for the use of the internet and social media.* This category was found in 329 cases. The most important reasons were communication (122/329), organization of daily affairs (55/329), and information search (53/329). Other causes related to job or study (45/329), entertainment (41/329), creativity (9/329), and pastime (4/329).

Baby Boomers put the most emphasis on communication, job/study, and information search. Entertainment was a minor issue for them, and while they organized their daily affairs online, they did not do so as often as the younger generations. A typical quote of one individual from the Baby Boomer group was,

In a conversation, I can ask Dr. Google or Wikipedia. At our last family reunion, I was very proud. Our city guide … said something, that and that. And I was so sure that this couldn't be right, and then I checked, and it was me who was right!

Baby Boomer, male, 64, group BB1

In contrast, Generation X reported the importance of the online organization of daily affairs even more often than communication, followed by information search. Job/study and entertainment was important to them to a certain extent, and this generation reported somewhat creative activities as a reason to be online. A typical quote of a Gen X member was,

I don't have an alarm clock either. Only the smartphone does that. Of course, I don't write letters anymore either… what for? There is mail for that. And WhatsApp groups are very, very important, also to keep in touch with the family. And, of course, apps, so there are apps like “sand by the sea.” No matter if ... if you ... so if I want to cook something, I get a recipe via app ...

Generation X, male, 48, group X1

This quote reflects the subcode “entertainment,” which was a relevant issue to this generation.

… what I like to do on the internet is watching YouTube videos. Different fail-videos from time to time, where people fall down and so, sometimes that is quite funny.

Generation X, male, group X1

The youngest generation, the Digital Natives, put the strongest focus on communication in comparison with the other groups, followed by entertainment and the organization of daily affairs. As well, they often look for quick information and use the internet for their study. In some cases, they use their mobile phones for mere pastime.

Well, I also use Instagram, but more when I'm bored or when I think, I've got about 15 minutes left before I have to leave the house and it's not worth the effort to start something. Then I scroll down a bit …

Digital Native, female, 20, group DN1

### Effects of the Use of the Internet and Social Media

The code *Effects of the use of the internet and social media* summarized discussion contributions that referred to the observed effects of the use of the internet and social media on the participants’ daily lives or on society. In total, 501 codings were found. The most important issues in this category were described by comparison of generations (100/501), followed by communication (92/501) and societal changes (77/501). Next, relevant aspects were simplification (54/501), outdated technology (eg, VHS; 43/501), language (27/501), and writing letters or postcards (25/501). Minor topics the participants talked about were financial issues (23/501), telephoning (20/501), loss of abilities (15/501), health (12/501), help/sharing (9/501), and environment (4/501).

The Baby Boomer groups placed emphasis on the comparison of generations, societal changes, and changes in communicative behavior. Further, they discussed outdated, formerly ”new“ technologies like VHS tapes, and they talked about writing letters or postcards, which they fear will become obsolete as well. They often observe changes in language.

In the WhatsApp group …sometimes I get the impression that I am the only one in my family who still knows something like punctuation.

Baby Boomer, 62, male, group BB1

Members of Generation X attached the highest importance to how new technologies simplified their daily affairs. In this context, they draw many comparisons between generations, talking about changes in communication and language. At the same time, they see a potential loss of abilities. Finally, financial issues seem important to them.

I also have my Payback card in my cell phone. I no longer have a plastic card. Download the app, and then you have a bar code and put it on their device, and then you have your Payback card.

Generation X, female, 51, group X1

While talking about the effects of the internet and social media, the Digital Natives had a tight focus on communication, societal changes, and comparisons of generations. They often talked about staying connected with members of the Baby Boomer generation, who were their parent generation. Further aspects were financial issues, health topics, and telephoning. Typical quotes regarding changes in communication referred to social relationships.

I think that maybe WhatsApp and etcetera have made friendships a bit deeper, that you just get a lot more from each other.

Digital Native, female, group DN1

Another quote illustrates the subcoding Comparison of generations in the context of communication as well.

I think it’s also a completely different writing style, how we write among each other and how I write with my parents. So, my mother just writes with dots and capitalization and so on. We really do not have that anymore.

Digital Native, female, 20 years, group Y1

### Personal Feelings and Experiences

The code *Personal feelings and experiences* were given in statements that expressed intimate observations of the participants regarding their emotions after or during the usage of the internet and social media. All groups expressed this in various forms, with 381 codings in total. The predominating subcategory was challenges (173/381), further differentiated into solitude, the feeling of permanent availability, norms and expectations felt by others, dissatisfaction, liability, time pressure, and acceleration. The second important domain of feelings were fears (123), which were expressed as fear of commercial interests, fear of surveillance, distrust and uncertainty, and feeling overstrained. Positive emotions (47) were expressed by talking about feeling anonymous, not having to go back to being without the internet, enjoying offline time, and curiosity and fascination. Some group members felt risks (31), especially the risk of addiction or loss of reality. Additionally, the participants showed indifferent emotions (7) (eg, by dismissing potential surveillance as unimportant). Further details are presented in Multimedia Appendix 2.

The feelings of the Baby Boomer generation were dominated by fears. Mistrust or feeling insecure regarding the quality of information provided by the internet and the fear of potential surveillance due to a lack of data security were expressed in many cases. Feeling overstrained and the fears of commercial interests were both topics of discussion.

The second important group of feelings related to challenges; the Baby Boomers often felt pressure from feelings of liability and being forced to stay permanently available with their mobile devices. Indifference was not shown at all. Positive emotions and risks were both present, but to a small degree.

I think something is slipping away, that's the danger … What actually happens to the data that I have entered there? Where do they end up? And what might be done with it?

Baby Boomer, female, 61, group BB2

Fears and challenges were expressed in the Generation X group as well. If they talked about fears, their concerns related to potential surveillance and mistrust or insecurity. Potential risks were felt by losing contact with reality. Positive emotions played the most important role in comparison to the other age groups. If positive, their emotions were described by the subcode curiosity and fascination.

Yes, but I find it exciting. I think that's interesting. And there you can make a data comparison, women my age, menopause and so on. You can just watch everything a bit. I think that's exciting.

Generation X, female, 51, group X1

The Digital Natives put the most emphasis on challenges that they experienced in the usage of the internet and social media. They felt pressured by the norms and expectations of others, such as the necessity to be online, to quickly respond to a message, and to be available all the time. If they expressed fears, these were related to potential surveillance, but in this group, some members voiced feelings of indifference at the same time. They accepted the fact of surveillance because they were aware that, otherwise, most functions would be useless if the terms of use of, for example, Facebook, were rejected. Positive emotions related to curiosity and fascination. Some members reported the feeling of risks regarding addiction or loss of reality. A typical example regarding norms and expectations are reflected in the following quote:

Yes, because it is such a social filter. Because everyone knows, he knows the rules of Instagram. I know I have to jazz everything up and somehow say, ”Wow, it's so cool what you posted there.“ So this is such a love culture there, isn't it? Where to exaggerate ...

Digital Native, male, 28, group DN2

### Self-relatedness

The main category, *Self-relatedness,* included expressions that showed an orientation towards person-based and individual choices. This code was used in all cases in which the behavior with the internet and social media was related to the individual (eg, to the surveillance of personal health, to self-presentation in a network or forum, or to personal organizing like calendars or diaries). In sum, 330 codings were found. Subcodes were distancing (114/330; ie, the conscious decision against being permanently available or following a specific internet trend), self-tracking (62/330), self-reflection (44/330), self-control (39/330), self-optimization (22/330), autonomy (11/330), and self-presentation (9/330). These aspects all referred to one’s own person. Self-relatedness observed in others (29/330) was also found in this domain.

The Baby Boomers expressed the least self-relatedness in comparison to the other generations, and they hardly talked about individual, person-based issues. They often expressed the wish to distance themselves from several developments of the internet and social media that they consider to be useless or even dangerous to them. Attempts of self-control refer to self-organization by using calendar apps or messaging to oneself, and self-reflection was sometimes done in an ironic way in the course of distancing from new technologies (eg, *”*I'm not ready for that yet*“*). If self-tracking was present, it was used for health care reasons. Self-optimization was no issue at all, nor was self-presentation in most cases.

I had a good feeling, for I could say, ‘'I won't join to do that” (regarding Facebook).

Baby Boomer, male, 64, group BB2

Regarding expressions of self-relatedness, Generation X also talked a lot about distancing, especially as far as self-presentation in platforms was concerned. Self-tracking was often done for several reasons; however, self-optimization was mostly not an issue, nor was the presentation of one’s own self in networks a matter of discussion. Self-reflection, seeking autonomy, and self-control measures were minor topics that referred to calendar apps, date reminders and notifications for self-organization, etc.

Yes, so I go running regularly. Do the Runtastic, which is also an app. But I have to say, what bothered me again, massively, that I could upload it to Facebook by pushing a button. So that everyone can see it, too. I thought that was stupid again.

Generation X, male, 47, group X1

Self-presentation was observed in others in a critical way.

It is a self-presentation. I was here on vacation, I have this, I have this, I have that, I was always eating, I have a lot of friends…

Generation X, female, 48, group X1

To the youngest generation, self-tracking was the most important subcategory in the context of self-relatedness. In tracking their behavior, sports activities or nutrition tracking were done, in many cases, explicitly for reasons of self-optimization. As well, the Digital Natives reported attempts to distance themselves from single forms of media usage; however, these attempts often fail. Self-reflection and self-presentation were a frequent issue of discussion as well.

I often notice that I am just grabbing it (the smartphone) relatively unconsciously and looking at something, … if you are walking alone through the city, in order to seem engaged … just take it and pretend to continue your education or something (laugh). That's quite ... a bit scary.

Digital Native, male, 21, group DN2

Another example shows the subcategory of self-relatedness observed in others, which was another common topic of discussion among the Digital Natives.

Many do this to compare themselves with friends. For example, I also know one who always posts that on Instagram, that she is the best, and that she walked most of the time or so (laugh) ... I think that's a bit silly. Posting such things is really just pushing yourself.

Digital Native, female, 22, group DN2

### Social Relationships

Statements that referred to online communication with others via the internet and social media (eg, with friends, colleagues, or family members) were titled *Social relationships.* In all generations, 214 codings referred to the main category *Social relationships.* The most often used subcode referred to social comparisons (70/214), followed by the relationship of the participants to their partners/friends (68/214) and family (53/214). Minor issues discussed in the groups were conflicts (8/214), gender stereotypes (6/214), associations/engagement (5/214), and social inequities (4/214).

The Baby Boomers described maintaining close relationships with their children or grandchildren with the help of their mobile devices. Staying connected to partners/friends was important to them as well. In this generation, few participants were members of the specific associations with which they were engaged. Social comparisons and conflicts were mostly not an issue. Gender stereotypes and social inequities were not discussed.

I'm in a WhatsApp group that only affects my ... core family, and since my 2 adult children have moved out, that's important, too, to maintain some social contact.

Baby Boomer, male, 62, group BB1

Members of the Generation X group cared for their relationships, especially with partners and friends. Social comparisons were often made. Only then, in third place, staying connected to family was a matter of discussion. Gender stereotypes and social inequities were not discussed, and conflicts and memberships in associations and respective engagement were discussed to a small degree. The following example illustrates the kind of contact a Gen X woman described with her friends:

There's a lack of personal contact; it has become less. In the past, you just took the phone or you met somebody, and today you quickly write a message. If it is someone's birthday, I avoid sending only a WhatsApp message; I actually call and congratulate.

Generation X, female, 44, group X2

The Digital Natives often addressed social comparisons directly and described their relationship to friends and partners as being shaped by online activities. In some cases, the Digital Natives talked openly about conflicts that emerged from misunderstandings due to social media messages. As well, potential gender stereotypes and social inequalities that manifest on Instagram were discussed.

For me personally, I find social comparisons much more difficult if they are people I know and ... if they post a picture every day, but I have to stay in the library, I think to myself, Okay, why do they manage that and I don't?

Generation Y, male, 28 years, group DN2

## Discussion

### Principal Findings

This study aimed to investigate the thesis of an ongoing individualization process [1,2,4] and how the internet and social media have contributed to this development. We looked for generational differences in the usage of the internet and social media that go along with emotional experiences and expressions of self-relatedness. We analyzed 6 age-specific focus group discussions, with 3 consecutive generations of Baby Boomers (born in 1950-1965), Generation X (born in 1966-1980), and Digital Natives (born in 1981-2000). If the ongoing individualization process is promoted by the use of the internet and social media, data should reveal that the generation of Digital Natives uses the internet in a more individualized, self-presenting, and self-optimizing way in comparison to older age groups. Moreover, if growing individualization can be attributed to the use of the internet and social media, we investigated which potential emotional and mental health consequences might be traced back to this development. Given the fact that psychological strain due to individualization cannot be observed directly, we regarded this issue by analyzing the expressions of feelings and experiences in the context of statements of self-relatedness.

Our findings suggest that the Digital Natives expressed more tendencies toward self-relatedness than the Baby Boomers, but less than the next recent generation, Generation X. The usage of self-tracking technology for fitness and health parameters was obvious in both younger generations. However, these 2 generations explained their choice for tracking technologies in a substantially different way. Generation X showed curiosity and fascination for the possibility of tracking personalized data, but not desires for self-optimization. In contrast, the Digital Natives reported making use of calorie-tracking apps or fitness apps with the aim of optimizing their own bodies. A previous study had investigated predictors of fitness and weight loss websites and found a close relationship between the internalization of beauty ideals, being female, and a high frequency of internet use in a population with a median age of 24 years [47].

Social networking played an important role for both younger generations but, again, for different reasons. While Generation X presented themselves as critical about posting vacation pictures or private information online, they were often members of Facebook or other social networking sites. Meanwhile, the Digital Natives were more inclined to present themselves regularly on microblogging-services like Instagram. They felt the need to stay visible and often reported observing this behavior in others.

Among Baby Boomers, the self-presentation of younger generations on social networking sites was observed with a sense of alienation. This generation expressed more reluctance and was not used to talking about personal issues in a group. In contrast, the Digital Natives talked openly about their personal entertainment, their fitness, and forthcoming events. This does not necessarily mean that the elders did not care about themselves, as this behavior might just be a communicative style. In another study carried out in a different cultural and communicative context (Hong Kong), similar generations were investigated regarding their social media use, and many facets of narcissism were found amongst Baby Boomers [24]. Future research could integrate both concepts: individualization as a kind of self-care on the one end of a potential continuum, and narcissism on the other end.

### Generation Profiles

As generations cannot be characterized entirely without expressions of self-relatedness, generation profiles regarding the characteristics of internet and social media use in each age group were used to contribute to a deeper understanding of the relationship between internet usage and individualization for each group. Also, the possible mental and emotional strains that may lead to a mental disorder must be reflected in each generation separately.

#### Baby Boomers

The Baby Boomers presented themselves as open-minded but were critical about their use of the internet and social media. Many of them used the internet as a “paperless newspaper” with a desktop computer or a smartphone, which was switched on and off at a certain time of the day. They appreciated the possibility of staying connected with their children and grandchildren but, at the same time, expressed feelings of distrust regarding the provenience of information or being overwhelmed by technical requirements. Baby Boomers are sometimes called “digital immigrants” [48] to describe the fact that many Baby Boomers have still not mastered computerization; for example, the use of emoticons is not common to every member of this generation [35]. Research on self-tracking of health-related data points in a similar direction: not all self-tracking is digital self-tracking. A substantial part of the self-trackers are “traditional” trackers (ie, do tracking manually by writing medical data on a sheet or in a booklet). While digital trackers are mainly men, young, and of higher income, the traditional trackers are often women, older than 55 years, and have lower income or are retired [29].

In our study, this generation aimed at autonomy and self-determination to a certain extent. Even when distancing themselves from certain unappreciated internet trends, they were concerned about their own development, often making person-based choices against the use of the newest trendy platform or messaging service. They wanted to stay connected to their own network and family members. Our result goes along with an earlier investigation of generational differences in social media networking virtual needs [48], which highlighted that the Baby Boomers especially, but also Generation X, show needs for competency and a sense of being effective. In regard to possible mental and emotional strains, our results show that while the Baby Boomers have to cope with the need to stay updated and feel the pressure of social-environmental expectations, they lack a deeper understanding of the online activities of their children and grandchildren. Psychosomatic research supports this observation: If still employed, Baby Boomers feel a high pressure in competing with younger colleagues [49]. Targeted coping strategies are needed for this generation, regardless of whether they are still employed or not.

#### Generation X

Our Generation X participants showed a great variety of internet and social media usage. This generation possessed the highest number of internet-capable devices per person, followed by the Baby Boomers and Digital Natives. Some members of this generation were technophiles (ie, curious to try wearable devices and used many apps to simplify their daily affairs). Consequently, the possibility of data surveillance was accepted as an unavoidable circumstance. This observation that Generation X is interested in forming their own identity in a positive, open-minded way was made by a cross-cultural study among German, Japanese, and US participants [36]. In our study, some of the Generation X group members appreciated personalized advertising, which is normally discussed as a harmful form of surveillance [50]. This reveals an inherent desire for personalized services geared toward the accommodation of individual wishes. Some participants presented themselves as rather restrictive due to fears of data surveillance. Nonetheless, they expressed this in a highly self-focused way by talking about concerns about their own health or regarding various risks, like the commercial interests of app developers.

Generation X reported being especially active on Facebook. Numerous studies have pointed to mental health, personality factors, and narcissism as predictors for the excessive use of Facebook. Several diverging results regarding the usage of Facebook show that a simple ”Facebook is harmful for mental health“ statement is not possible. A meta-analysis found associations between the use of social networking sites like Facebook and depression. The authors show that this association can be explained by the users' social comparisons and not by the mere time spent on the sites [51], which hints at the same underlying mechanisms found in our study. Other studies reveal that the correlation of depressive symptoms with the excessive use of Facebook can be explained with high degrees of neuroticism [26]. Facebook users, in comparison to nonusers, tend to show more narcissism and self-esteem, as well as higher degrees of life-satisfaction [23]. Resilience factors like self-esteem and curiosity should be strengthened in this generation while taking data surveillance fears seriously.

#### Digital Natives

The youngest generation, the Digital Natives, were not always as self-focused as they are reputed to be in a previous German survey study [34]. Satisfied with owning a smartphone alone and sometimes a notebook, they expressed intentions of adequately communicating with their parents or grandparents' generation. Additionally, they showed a high level of reflection about their own behavior on the internet and social media. They were critical observers of others' self-presentation, especially regarding the use of platforms like Instagram or Snapchat.

It is noteworthy that the youngest generation felt significantly more pressured by the norms and expectations of their social environment (eg, by having to answer immediately to a text message, staying on Instagram every few hours, or posting personal updates regularly). Furthermore, they drew many social comparisons based on information delivered by the internet (”My colleagues are having fun; I have to study, and this means that there is something wrong with me“). Not surprisingly, ”enjoying offline time“ was a particular matter of discussion in this generation.

### Future Implications

Social comparisons are a natural part of social exchange and play an important role in identity formation and thus are a necessary step in growing up and reaching adulthood [9]. The Digital Natives in our study were adults but may still be undergoing identity formation, which, according to the renowned psycho-analytical researcher, Erikson [11], is a life-long process. The question arises, then, as to whether the comparisons felt by Digital Natives are a precondition or a result of their identity formation. Ultimately, the immediacy of information about the social activities delivered via social media has the potential to cause a continuous, subtle strain on the identity of the younger generation, which is cross-linked by different types of technology. This does not need to end up in dramatic cyberbullying scenarios, which are often reported among adolescents [52,53], but may lead to increased mental distress for people vulnerable to mental health problems.

Studies that concentrate on patients with mental disorders show contradictory results. Patients with mental health disorders feel negatively affected by social media applications but believe that health care apps and calendars can positively influence their mental health [54]. However, patients with severe mental illnesses reportedly benefited from social media usage, which helped them participate in social life [55]. New methodological instruments like the social media disorder scale have emerged and will provide a more differentiated view on the topic [56].

Previous research on mental health and social media tended to focus on the apparent and strong effects of internet addiction due to extensive gaming [21] or eating disorders [57]. There is a strong need to promote age-specific investigations on more subtle associations, like performance pressure due to social comparisons among younger generations, or feelings of mistrust and overwhelm among older generations. The categories found in this study might serve as a template for the development of age-specific questionnaires after integrating further results of other cultures. This study design could be part of an exploratory design, which normally starts with an in-depth qualitative analysis and leads to a broader, epidemiologic perspective [58].

### Limitations

The definition for age groups of specific generations varies significantly in the literature. Hurrelmann [34], for example, defines a generation in 15-year periods, with Generation X starting in 1970 and ending in 1985. While other studies agree that this generation started in 1965, the ending year ranges from 1976 [24], 1978 [36], 1982 [33], and even to 1983 [48]. However, we used the definitions provided by Palfrey and Gasser [34] regarding Digital Natives and went back in increments of 15 years, which may or may not be the universally accepted age groups.

This study followed all principles of good scientific practice; however, the most challenging limitation was the issue of age effects versus cohort effects, which is a recognized problem in social sciences. As Schröder [59] remarked, it is not possible to carve out differences between generations without controlling for age as a variable. This problem might only be resolved by choosing a longitudinal study design.

Moreover, the qualitative coding and interpretative work were done by the author alone, so no interrater reliability can be provided. In addition, our participants were recruited in a predominantly academic environment at the University of Heidelberg in Germany; it is understood that these people may use the internet and their social networks in more ambitious ways than might be generalizable to people of other professions or countries.

### Conclusions

The Digital Natives and Generation X participants expressed more individualistic behaviors, like self-tracking, self-optimization, and self-presenting in social networks, than Baby Boomers. They also often observed self-presenting behavior in friends and colleagues. In contrast, the Baby Boomers were less driven by individualization but seemed very interested in taking advantage of the possibility of staying informed and connected while not following every new technological trend. They kept a critical distance from the internet and social media but also did not want to be left behind the developments.

The most striking difference among the generations was the high pressure of norms and expectations of the social environment and the social comparisons felt by the Digital Natives. It remains unclear if this is a sign or a precondition of individualization, but it is known that social comparisons may result in mental distress. Further investigations on this association are necessary to promote a mentally healthy way of using the internet and social media.

## Appendix

Multimedia Appendix 1 Summary of the main categories and subcategories used for the focus groups.

Multimedia Appendix 2 Inductive codes and subcodes in the generations (total frequencies).
