# Supplementary material for: Expressions of Individualization on the Internet and Social Media: Multigenerational Focus Group Study
Source: J Med Internet Res. 2020 Nov 4;22(11):e20528. doi: 10.2196/20528 (PMC7673976; doi:10.2196/20528)
Supplement: Multimedia Appendix 1 [file jmir_v22i11e20528_app1.docx]

| **Main codes** | **Definition** | **Subcodes** | **Supporting quotes** |
| --- | --- | --- | --- |
| Reasons for the use of internet and social media | Expressions of individual reasons and causes for the use of the internet and social media | Communication | *"… what I like to do on the internet, is watching Youtube videos. Different fail-videos from time to time, where people fall down and so, sometimes that is quite funny." (Generation X, male, 47, group X1)*  *"Well, I also use Instagram, but more when I'm bored or when I think: I've got about fifteen minutes left before I have to leave the house and it's not worth the effort to start something. Then I scroll down a bit …" (Digital Native, female, 20, group Y1)* |
|  |  | Creativity |  |
|  |  | Entertainment |  |
|  |  | Information search |  |
|  |  | Job, study |  |
|  |  | Organization of daily affairs |  |
|  |  | Pastime |  |
| Effects of the use of internet and social media | Expressions that reflect general effects caused by the internet and social media, which may be observed or expected; except effects on personal feelings and experiences | Communication | *"When you think about the census in Germany: What a huge tussle did we have! We elder remember it, in those days we thought: This is the end of the world or whatever. I cannot even say, whether this was justified or not. But today all these things are okay." (Baby Boomer, male, 62, group BB1)*  *"I think it’s also a completely different writing style, how we write among each other and how I write with my parents. So, my mother just writes with dots and capitalization and so on. We really do not have that anymore." (Digital Native, female, 20, group Y1)* |
|  |  | Comparison of generations |  |
|  |  | Environment, Ecology |  |
|  |  | Financial issues |  |
|  |  | Health |  |
|  |  | Help, sharing |  |
|  |  | Language |  |
|  |  | Loss of abilities |  |
|  |  | Outdated technology (i.e. VHS) |  |
|  |  | Simplification |  |
|  |  | Societal change |  |
|  |  | Telephone |  |
|  |  | Writing letters or postcards |  |
| Personal feelings and experiences | Expressions of personal feelings and emotions regarding the own usage of the internet and social media and experiences with the issue in general | Challenges (solitude, availability, norms and expectations, dissatisfaction, liability, time pressure and acceleration) | *"You can spend hours on it without finding a result, because sometimes it is not so targeted, or because you are also directed to pages, which you then open, of course, but then you realize: 'I can't get any further with that.'" (Baby Boomer, female, 56, group B1)*  *I've become a lot more impatient. Because you have the opportunity to quickly exchange information within seconds, it often happens to me that I then write a message again, or instead of waiting until the phone call with the mom in the evening, then again I just quickly wrote a WhatsApp.*  *(Digital Native, female, 22, group Y1)* |
|  |  | Fears (fear of commercial interests, fear of surveillance, distrust and uncertainty, overstraining) |  |
|  |  | Indifference (acceptance of surveillance) |  |
|  |  | Positive emotions (feeling anonymous, never again without internet, enjoying offline time, curiosity and fascination) |  |
|  |  | Risks (loss of reality, addiction) |  |
| Self-relatedness | Expressions that show an orientation towards person-based and individual choices, which may promote the own progress (e.g. self-optimization) or presentation towards others (self-presentation) or may be supported by technology (self-tracking) | autonomy, self-determination | *"For that day I had a good feeling, for I could say: 'I won't join to do that" (Note: Facebook) (Baby Boomer, male, 64, group B2)*  *"I have a fitness tracker and I really dig it. Well, I won't always scan myself from head to toe, but the steps are interesting, and observing the sleep I find totally exciting, because you often cannot quite judge for yourself ... Did I really sleep well now? Or why am I tired now? I think that's cool." (Generation X, female, 51, group X1)*  *"I often notice that I am just grabbing it (remark: the smartphone) relatively unconsciously and looking at something, … if you are walking alone through the city, in order to seem engaged … just take it and pretend to continue your education or something (laugh). That's quite ... a bit scary."* (Digital Native, male, 21, group Y2) |
|  |  | distancing (to differentiate oneself from actual developments in the internet) |  |
|  |  | self-control (i.e. using an app for controlling hours spent online) |  |
|  |  | self-optimization |  |
|  |  | self-presentation |  |
|  |  | self-reflections (i.e. expressing very personal observations like being an internet junky or feeling overwhelmed) |  |
|  |  | self-relatedness observed in others |  |
|  |  | self-tracking (i.e. using fitness tracker or wearable devices) |  |
| Social relationships | Expressions describing relationships to their own social environment | Associations, engagement | "I'm in a WhatsApp group that only affects my ... core family, and since my two adult children have moved out, that's important, too, to maintain some social contact." (Baby Boomer, male, 62, group BB1)  "For me personally, I find social comparisons much more difficult, if they are people I know and ... , if they post a picture every day but I have to stay in the library, I think to myself: 'Okay, why do they manage that and I don't?'" (Generation Y, male, 28, group Y2) |
|  |  | Conflicts |  |
|  |  | Family, relatives |  |
|  |  | Gender stereotypes |  |
|  |  | Partners, friends |  |
|  |  | Social comparisons |  |
|  |  | Social inequities |  |
