# Supplementary material for: Expressions of Individualization on the Internet and Social Media: Multigenerational Focus Group Study
Source: J Med Internet Res. 2020 Nov 4;22(11):e20528. doi: 10.2196/20528 (PMC7673976; doi:10.2196/20528)
Supplement: Multimedia Appendix 2 [file jmir_v22i11e20528_app2.docx]

| **Codes** | **Subcodes** | **Generation** | | | |
| --- | --- | --- | --- | --- | --- |
|  |  | Baby Boomer (n = 11) | Generation X  (n = 10) | Digital Natives  (n = 15) | Sum  (N = 36) |
| Reasons for the use of internet and social media | |  |  |  |  |
|  | communication | 31 | 31 | 60 | 122 |
|  | creativity | 1 | 5 | 3 | 9 |
|  | entertainment | 6 | 12 | 23 | 41 |
|  | information search | 18 | 21 | 14 | 53 |
|  | job, study | 19 | 15 | 11 | 45 |
|  | organization of daily affairs | 6 | 33 | 16 | 55 |
|  | pastime | 0 | 1 | 3 | 4 |
|  | **sum** | **81** | **118** | **130** | **329** |
| Effects of the use of internet and social media | |  |  |  |  |
|  | communication | 25 | 36 | 31 | 92 |
|  | comparison of generations | 39 | 37 | 24 | 100 |
|  | environment | 0 | 4 | 0 | 4 |
|  | financial issues | 1 | 11 | 11 | 23 |
|  | health | 0 | 4 | 8 | 12 |
|  | help, sharing | 1 | 7 | 1 | 9 |
|  | language | 13 | 12 | 2 | 27 |
|  | loss of abilities | 2 | 12 | 1 | 15 |
|  | outdated technology (e.g. VHS) | 22 | 19 | 2 | 43 |
|  | simplification | 6 | 41 | 7 | 54 |
|  | societal change | 29 | 23 | 25 | 77 |
|  | telephoning | 4 | 8 | 8 | 20 |
|  | writing letters or postcards | 11 | 12 | 2 | 25 |
|  | **Sum** | **153** | **226** | **122** | **501** |
| Personal feelings and experiences | |  |  |  |  |
|  | challenges\acceleration, time pressure | 7 | 3 | 8 | 18 |
|  | challenges\availability | 11 | 2 | 9 | 22 |
|  | challenges\dissatisfaction | 2 | 9 | 6 | 17 |
|  | challenges\liability | 18 | 8 | 34 | 60 |
|  | challenges\norms and expectations | 4 | 13 | 37 | 54 |
|  | challenges\solitude | 2 | 0 | 0 | 2 |
|  | challenges (sum) | 44 | 35 | 94 | 173 |
|  | fears\ distrust and uncertainty | 21 | 10 | 1 | 32 |
|  | fears\ fear of commercial interests | 7 | 7 | 7 | 21 |
|  | fears\ fear of surveillance | 18 | 27 | 16 | 61 |
|  | fears\ overstraining | 7 | 2 | 0 | 9 |
|  | fears (sum) | 53 | 46 | 24 | 123 |
|  | indifference | 0 | 1 | 6 | 7 |
|  | positive emotions\ curiosity and fascination | 8 | 23 | 5 | 36 |
|  | positive emotions\ enjoying offline time | 1 | 2 | 3 | 6 |
|  | positive emotions\ feeling anonymous | 2 | 0 | 0 | 2 |
|  | positive emotions\ never offline again | 0 | 1 | 2 | 3 |
|  | positive emotions (sum) | 11 | 26 | 10 | 47 |
|  | risks\addiction | 4 | 2 | 6 | 12 |
|  | risks\loss of reality | 5 | 6 | 8 | 19 |
|  | risks (sum) | 9 | 8 | 14 | 31 |
|  | **Sum** | **117** | **116** | **148** | **381** |
| Self-relatedness | |  |  |  |  |
|  | autonomy, self-determination | 5 | 5 | 1 | 11 |
|  | distancing | 43 | 48 | 23 | 114 |
|  | self-control | 17 | 13 | 9 | 39 |
|  | self-optimization | 0 | 2 | 20 | 22 |
|  | self-presentation | 2 | 2 | 5 | 9 |
|  | self-reflection | 8 | 18 | 18 | 44 |
|  | self-tracking | 9 | 26 | 27 | 62 |
|  | self-relatedness observed in others | 0 | 7 | 22 | 29 |
|  | **sum** | **84** | **121** | **125** | **330** |
| Social relationships | |  |  |  |  |
|  | associations, engagement | 3 | 2 | 0 | 5 |
|  | conflicts | 1 | 3 | 4 | 8 |
|  | family, relatives | 23 | 10 | 20 | 53 |
|  | gender stereotypes | 0 | 0 | 6 | 6 |
|  | partners, friends | 12 | 26 | 30 | 68 |
|  | social comparisons | 3 | 24 | 43 | 70 |
|  | social inequities | 0 | 0 | 4 | 4 |
|  | **sum** | **42** | **65** | **107** | **214** |
| **Total sum** | | **477** | **646** | **632** | **1755** |
